# Seasonal variations and temporal instability of motorcyclist injury severity in Cambodia: Analyses based on a random parameter logit model with heterogeneity in means and variances

**DOI:** 10.1016/j.heliyon.2024.e39722

**Published:** 2024-10-22

**Authors:** Yaqiu Li, Junyi Zhang, Haoran Li, Yunpeng Lu, Lon Virakvichetra

**Affiliations:** a School of Transportation, Southeast University, Nanjing, 211189, China; b Graduate School of Advanced Science and Engineering, Hiroshima University, Higashi Hiroshima, 739-8529, Japan; c School of Automobile and Traffic Engineering, Wuhan University of Science and Technology, Wuhan, 430065, China; d Suzhou Automotive Research Institute, Tsinghua University, Suzhou, 215299, China

**Keywords:** Motorcycle crash, Injury severity, Seasonal variation, Heterogeneity in means and variances, Developing country

## Abstract

Motorcycles are a prevalent mode of transportation in countries like Cambodia that experience distinct rainy and dry seasons. However, the safety concerns associated with motorcycling in this region have not been thoroughly investigated. This study addresses this research gap by examining the severity of motorcyclist injuries in Cambodia, considering the potential variations across seasons and the fluctuations in contributing factors over time. Utilizing a random parameter logit model with heterogeneity in means and variances, the research analyzes motorcycle crash data from 2015 to 2017 to identify heterogeneities in the determinants of injury severity. The study confirms seasonal variations and temporal instabilities in influential factors, highlighting the need for distinct modeling for dry and rainy seasons due to varying contributing factors. Key findings include the consistent increase in fatal injury risk associated with head-on collisions and elderly rider involvement across both seasons. During the rainy season, motorcycle-to-motorcycle crashes significantly heighten the likelihood of severe injuries, with weekend crashes more likely to result in fatalities. Furthermore, more than half of speeding incidents during the rainy season consistently led to fatal injuries across all three years. In contrast, during the dry season, riders faced a greater risk of severe injuries compared to pillion riders, with crashes on national roads more likely to lead to fatal outcomes. Temporal stability tests further reveal that the influence of external variables on motorcyclist injury severity varies across years, stressing the need for tailored, season-specific approaches to effectively mitigate and prevent crashes.

## 1 Introduction

Every year, nearly 50 million people are injured in roadway crashes around the world, with as many as 1.3 million others lost their lives; over half of these fatalities occur among vulnerable road users like motorcyclists, pedestrians, and cyclists [1]. Compared to four-wheeled car crashes, motorcyclists face limited protective measures against injury, making them significantly more vulnerable to sustaining injuries [2,3]. For every mile traveled, motorcyclists face a mortality risk approximately 29 times higher than that of occupants in passenger cars [4]. Over 90 % of the motorcyclist deaths occur in low-income and middle-income countries, and the crash death rate is over three times higher than that of high-income countries [1]. Among the developing countries of Southeast Asia, Cambodia has the highest road safety concerns [5], recording 7.0 fatalities per 10,000 registered vehicles, which exceeds the rates in Lao PDR2 (5.8) and Vietnam (1.8) [6]. In Cambodia, traffic crashes remained the leading cause of mortality [7], and annual motorcycles fatalities are more than 1200 from 2011 to 2019, consistently accounting for over 75 % of total traffic fatalities, as well as significantly outnumbering four-wheeled vehicles [8].

In developing countries like Cambodia, where motorcycle dominant as the main transportation mode, motorcycles and tricycles are involved in over half of these fatalities, highlighting the urgent need for stricter safety regulations and policies regarding motorcycle and tricycle use in these countries [5]. The growing road safety issues in Southeast Asia are strongly linked to the significant increase in motorcycle and tricycle prevalence in the past two decades, mainly due to their affordability compared to four-wheeled cars. Despite their vulnerability, motorcyclists often receive inadequate attention in road safety efforts. While an increasing amount of research has explored the risk factors and patterns of motorcycle crashes, to the best of the authors’ knowledge, there is a lack of research on motorcyclist injury severity in Cambodia, particularly concerning the distinct impacts of rainy and dry season.

Regarding the heterogeneity in seasonal effect on motorcyclist injury severity, numerous research has claimed that weather conditions are strongly correlated with traffic crash severity due to poor visibility and reduced friction from slippery road conditions [9,10]. Cambodia lies within the tropical monsoon climate zone, with high temperatures and seasons divided into rainy and dry periods [11]. During the rainy season from May to November, heavy rainfall is found across the country with the average annual precipitation is 1400–2000 mm [12]. On the other hand, during the dry season from December to April, there is little precipitation, and the average monthly precipitation is about 10 mm in January [8,13]. In Cambodia, floods cause the largest scale of damage, followed by drought [14]. Due to these climate disasters and lower level of paved roads in Cambodia, poor visibility because of the dust in dry season and road disruption in rainy season adversely impact the lives of residents and logistics of local areas [15,16]. People's activities change depending on rainy and dry season make it is necessary to take into account the seasonal patterns of traffic fluctuations [17]. Studies also show the seasonal patterns, risk factors may change over time, thus leading to varied influence on injury severity [18]. Given this stark contrast, the importance of studying the factors contributing to rainy and dry season motorcycle crashes and their temporal instability in developing countries, particularly Cambodia, cannot be overstated.

To fill in the above research gap, this study focuses on determining the factors that impact motorcycle injury severity in Cambodia, with particular consideration of seasonal and temporal variations. Following this analysis, the paper will propose informed policy recommendations to mitigate existing motorcycle safety issues. It will begin with the motorcycle crash literature review, followed by a presentation of the methodology and a description of the dataset. Following that, we offer transferability testing, a detailed explanation of the estimation results from the random parameter multinomial logit model, along with the implications of the findings, and conclude with a summary.

## 2 Literature review

### 2.1 Research on motorcycle crashes

Previous research on motorcycle crashes and injury severity outcomes are summarized in Table 1. Injury severity, a crucial indicator when modeling crash data, is often treated as a discrete variable with severity levels such as property damage only (PDO), minor injury severity (MI), severe injury severity (SI), and fatal injury severity (FI). The literature review on motorcycle crash severity across these studies shown in Table 1, which highlights several critical factors, especially in low- and middle-income regions. High-speed driving and aggressive behavior consistently emerge as significant risks in India, Myanmar, and Pakistan. The non-use of helmets is another major factor contributing to higher injury severity, as noted in studies from Nigeria, Vietnam, and China. Alcohol impairment before riding further elevates the risk of severe injuries, a common theme in research from Laos, Ghana, and Vietnam. Lack of riding experience and proper licensing are prominent in increasing crash severity, with notable mentions in Myanmar, Vietnam, and China. Wet and slippery roads, common during the rainy season, are significant hazards, as seen in Kenya and India. Riding at night or in low visibility conditions, often worsened by monsoon weather, also leads to higher injury severity, highlighted in studies from Pakistan and China. And these studies also indicate that the factors influencing motorcyclist injury severity may vary over time in response to rider characteristics, road conditions, and other temporally changing variables [3,[19], [20], [21]].

**Table 1:** Summary of previous research on motorcycle crash.

Authors	Methodology	Data	Factors increase the likelihoods of more severe injury
Zambon and Hasselberg, 2006 [22]	Binary logistic regression	Swedish	High-speed driving behaviour; break the law and violate the rules of safe riding.
Rathinam et al., 2007 [23]	Multivariate logistic regression	Yamunanagar, India	The children rider who has aggressive behaviour and encounters with police; high-speed driving behaviour; tailgating.
Pai and Saleh, 2008 [24]	Ordered probit models	Britain	Male or elderly rider; heavier motorcycle; more than three vehicles were involved in collisions; collisions with heavier vehicles (bus/coach or HGV).
Majdzadeh et al., 2008 [25]	Multiple logistic regression	Qazvin-Loshan Road, Iran	Female, not using safety equipment; collision severity; crashes involving motorcycles and other vehicles; inclement weather conditions; ejection following collision and multiple collisions.
Haque et al., 2010 [26]	Hierarchical poisson models	Singapore	More lanes; roads with a wide median; speed limit ≥50 km/h.
Huang and Lai, 2011 [27]	Cox regression models	Taiwan, China	Drunk driving; advanced age; collisions with trees; driving at night; curved roads; driving on local roads.
Keall and Newstead, 2012 [28]	Logistic model	New Zealand	Rural road; bad road; pavement ridges.
Rifaat et al., 2012 [29]	Ordered logit model; heterogeneous choice model; partially constrained generalized ordered logit model	Calgary, Canadian	Speeding; right angle crashes; crashes during peak hours; left-turn-across-path crashes.
Lwin and Aung, 2012 [30]	Logistic regression analysis	Magway township, Myanmar	Under 25 years old; <1 year driving experience; alcohol; speeding; nighttime; highway.
Moskal et al., 2012 [31]	Multivariate analysis	France	Drunk driving; novice rider; male; driving without a license; during travel; speeding.
Haque et al., 2012 [32]	Log-linear models	Singapore	Collisions on the middle lane; intersection without signal lights.
Bjørnskau et al., 2012 [33]	Logistic regression model	Norway	Lack of experience; young age; speeding; alcohol; drugs.
Shaheed et al., 2014 [34]	Random parameter model	Iowa, USA	Rider inattention to the road; dry roadway surface; night; clear weather; male; in multi vehicle and motorcycle collisions involving large vehicles; without a helmet.
Jones et al., 2013 [35]	Straightforward multinomial logit model	Alabama, USA	Drunk driving; speeding; aggressive operation; weekend; collisions with passenger vehicles; collisions with large vehicles; clear weather; roadway curvature.
Blackman and Haworth, 2013 [36]	Ordered probit regression model	Queensland, Australia	Single vehicle; speed >80 km/h; curves; weekends; night time; elderly rider.
Jung et al., 2013 [37]	Multinomial logit model	California, USA	Victim ejection; alcohol or drug effects; collisions (including head-on, broadside, hit-object types) with truck involvement; weekend.
Chung et al., 2014 [38]	Ordered probit model	Seoul, South Korea	Crashes of drunk motorcyclists; crossing a central line; crashes with trucks; broadside crashes between motorcycle and vehicle; crashes during the night; high crash speed.
Maistros et al., 2014 [39]	Mixed logit model	Ohio, USA	Drunk driving; presence of passengers; curves; collision with sturdy roadside objects (such as guardrails and pillars); the increase in rider age.
Matheka et al., 2015 [40]	Descriptive statistics	Kenya	Rider's negligence; wet and slippery road surface caused by rain or sand; speeding; night; not wearing protective equipment (reflective clothing and helmet).
Bambach and Mitchell, 2015 [41]	Logistic regression model	Australia, USA	Drunk driving; without helmet; speeding; collision with trees and posts/poles.
Slesak et al., 2015 [42]	Multivariate logistic regression models	Luang Namtha, Lao	Young rider; male; alcohol; evenings; no helmet use; New Year; no rider license.
Oluwadiya et al., 2016 [43]	Logistic regression analysis	Nigeria	Carrying more than 1 passenger; no helmet use; young rider; riding too fast; riding without a license.
Chang et al., 2016 [44]	Mixed ordered logit model	Hunan, China	Over 60 years old; no helmet use; colliding with a heavy motorized vehicle in darkness; speeding; intersections with signal lights.
Lili et al., 2016 [45]	Multiple logistic regression	Shantou city, China	Lack of safeguard precautions; male; during the time of 0:00–6:00 and 18:00–24:00; young rider; rural area.
Wang et al., 2016 [46]	Ordered logit model	USA	Rider; uneven roads; adverse weather; slippery road surfaces; visual obstructions; alcohol; failure to wear a helmet; dark roads without lighting; curvy roads.
Islam and Brown, 2017 [47]	Random parameter logit models	Alabama, USA	Older motorcycle riders; no helmet use; collisions on highways; roadway horizontal curve; accidents on dark roads without lighting.
Xin et al., 2017 [48]	Mixed-effects logistic model	Florida, USA	Sharp curves (radius <1500 ft); older riders; reverse curves; high-speed (>50 mph); vegetation medians and paved shoulders; dry road surfaces; darkness; weekends.
Chung and Song, 2018 [49]	Nonlinear canonical correlation analysis	Seoul, South Korea	Age (motorcyclists in their teens and over fifty years old); speed over 30 km/h; crashes with heavy vehicles; collisions occur on roads narrower than 6 m; on curved sections; head-on collisions.
Das et al., 2018 [50]	Deep learning	Louisiana, USA	Rider ejection; two-way roadways with no physical separation; curved roads; weekends; young rider.
Pai et al., 2018 [51]	Logistic regression model	Taiwan, China	No helmet use; female and elderly riders; rural roads; unlicensed rider; alcohol consumption; single-vehicle crashes.
Doan and Hobday, 2019 [52]	Multiple logistic regression model	Ho Chi Minh City, Vietnam	Unlicensed motorcyclists; motorcycle crashes occurred between 6 p.m. and 12:00 a.m.; darkness; no helmet use; speeding; using mobile phones during riding.
Waseem et al., 2019 [2]	Random parameters logit model	Rawalpindi city, Pakistan	Riders without education; riders aged 25–50 years; collision of a motorcycle with a passenger car and fixed object (barrier, curb-stone, pole); riding on roads with a speed limit of 70-kmph or higher; Crashes that occur on weekdays; increased engine capacity.
Wali et al., 2019 [53]	Fixed and correlated random parameter Tobit models	Orange County, California, USA	Drunk riding; drug; elder rider; riders who had accidents on highways without a median and on flat terrain.
Chang et al., 2019 [54]	Latent class cluster analysis; Random parameters logit model	Hunan, China	Rural area; drunk riding; no helmet use; riding without a license; turn left; first-class; second-class and lower highways; visibility lower than 50 m; twilight; complete darkness; time between 20:00–23:59.
Abrari Vajari et al., 2020 [55]	Multinomial logit model	State of Victoria, Australia	Rider aged over 59 years; weekend; midnight/early morning crashes; multiple vehicles involved in the crash; t-intersections; crashes in towns and rural areas; intersections; roundabouts.
Kashani et al., 2020 [56]	Structural equation model	Iran	Collision with a heavy vehicle; old rider; daytime; dry road surface; rural roads; lack of road shoulder; two-way roads.
Hu et al., 2020 [57]	Classification tree model; Logistic regression model	Ningbo, China	Over 60 years old rider; male; intersection; rainy; cloudy/foggy; speed over 30 km/h.
J. Li et al., 2021 [58]	Latent class clustering; Ordered probit models	Orange County, California, USA	Multiple vehicle collisions near intersections; crashes occurred on vertical alignment for single-vehicle crashes and multi-vehicle crashes; rider with permanent physical impairments.
X. Li et al., 2021 [59]	Geographically-Temporally weighted ordered logistic regression	Pennsylvania, USA	No helmet use; abnormal driving condition; lack of safety training; large engine size; long motorcycle age; rider over 55 years old; speeding; mountainous road segments; head-on collisions.
Sivasankaran et al., 2021 [60]	Ordered logit model	Tamilnadu, Indian	Collision with fixed objects; run-off-road crashes; hitting equipment; debris on the roadways; winter; urban areas; older rider; holding probations or expired license; inclement weather conditions.
Islam, 2021 [19]	Random parameter logit models with heterogeneity in means and variances	Florida, USA	Motorcyclist under 30 with 10 m/h over the speed limit; weekend; during midnight (12 a.m.) and 6 a.m.; Motorcyclists aged 30–49 with collisions involving fixed objects; no helmet use; left-curved roadways; dark; speeding; crashes with normal driving condition; Motorcyclist aged 50 and above with crashes between 12 p.m. and 3 p.m. (off peak time); left curved roadway; no helmet use; collisions involving fixed objects.
Santos et al., 2021 [61]	Ordered logistic regression	Portugal	Rest days; clean and dry roads; rural areas; bended roads; national roads; male; no helmet use; blood alcohol content between 0.5 g/L and 0.8 g/L; truck involvement.
Pervez et al., 2021 [62]	Binary logit models (fixed and random parameter)	Pakistan	Weekend; summer; morning (06:00–08:59); nighttime (20:59–05:59); young riders (age ≤24); elderly riders (age ≥55); a multi-vehicle motorcycle crash involving heavy vehicles.
Zubaidi et al., 2022 [20]	Random parameter logit models with heterogeneity in means and variances	Florida, USA	At the intersection: speeding, hitting a school bus; darkness; 6 a.m.–12 p.m.; downhill; two-way roads; paved shoulders; no traffic control; At non-intersection: speeding; lane departure; front-front crash; hitting a heavy truck and commercial vehicles; darkness; morning; curved (horizontal/vertical) roads.
Chang et al., 2022 [21]	Random parameters generalized ordered probit model with heterogeneity in means	Hunan Province, China	Horizontal curves; the roads with high-speed limits 60–100 km/h; unlighted darkness; angular crashes; single-vehicle crashes; collision with heavy motorized vehicles; riders over 59 years old; accidents in rural areas.
Li et al., 2022 [63]	Multinomial logit model; Ordered logit model	Hunan Province, China	Unlicensed riding; alcohol-impaired riding; improper overtaking/lane changes; motorcyclists aged over 61; male; no helmet use; nighttime riding in the absence of streetlights.
Tamakloe et al., 2022 [64]	Binary logit regression model; Association rule mining	Accra, Ghana	At signalized intersections: daylight condition; right-angle; shoulder and median presence; motorcyclists with full license status; weekday. At non-signalized intersections: right-angle; casualty age less than 30; median presence; shoulder absence.
Chen and Mu, 2022 [65]	Multilevel logistic regression model	Taiwan, China	Male; old rider; no valid license; drunk driving; no helmet use; turning or overtaking others; early morning and evening riding; errors in traffic signaling; speeding.
Wang et al., 2022 [3]	Random parameter logit model with heterogeneity in means and variances	Rawalpindi, Pakistan	For male motorcyclists: young riders (below 20 years of age); collisions with another motorcycle; speeding; collisions with passenger car; 50 years and above riders; distracted driving; For female motorcyclists: distracted driving; collision of two motorcycles; U-turns crashes; weekday; riders above 50 years; speeding.

Varied discrete regression methods have been used to examine the effect of contributing factors on injury severity [66]. Multinomial probit/logit frameworks were employed when treating injury severity as a non-ordinal outcome variable, and ordered logit frameworks were used when treating injury severity as an ordinal outcome variable [67]. However, it is evident that both multinomial probit/logit frameworks and ordered logit frameworks are fixed parameter models that assume constant parameters across all observations. This assumption can lead to biased and erroneous estimation results [68,69]. To address this issue, transportation safety researchers have introduced the concept of unobserved heterogeneity to enhance estimation accuracy [70]. By considering unobserved factors that may influence the outcome, researchers can obtain more accurate and robust estimates for the effects of contributing factors on injury severity in motorcycle crashes.

### 2.2 Research on monsoon climate

Transportation infrastructure, designed to be resilient against weather, faces challenges due to climate change over time [71]. Phenomena like sea level rise, altered precipitation patterns, extreme weather events, and increased heat can disrupt transportation networks, strain infrastructure, and create safety hazards. Heavy rainfall may lead to floods and mudslides, damaging roads, bridges, and other transport systems, hindering access to essential services and prompting shifts in transportation methods, thereby causing congestion and heightened risk of traffic accidents. Conversely, drought conditions, especially when coupled with extreme heat, can heighten wildfire risks, which in turn can damage transport routes and reduce visibility for motorcyclists. The impact of these changes varies regionally – for instance, blocked roads in one area might lead to congestion elsewhere. Similarly, fluctuating water levels in certain regions can influence transportation networks in other areas. Furthermore, the transportation sector is interconnected with other economic sectors like water resources and energy, meaning climate change effects on these sectors can also impact transportation systems [72].

However, most of Southeast Asian countries like Cambodia, Myanmar, Vietnam, Laos, Thailand, Indonesia, Malaysia, the Philippines etc., experience a monsoonal climate characterized by distinct rainy and dry seasons. Various studies have demonstrated that weather conditions and monsoon patterns have a substantial effect on travel behavior and the frequency of traffic crashes in Southeast Asian nations [13,15,16,[73], [74], [75], [76]]. In Cambodia, where motorcycle crashes have emerged as a serious issue in the region due to the rapid increase in motorcycle registrations without adequate management measures and the lack of sufficient protective measures for motorcyclists, the effects of the rainy and dry season extend beyond weather patterns and impact road safety and traffic crashes [3,77], particularly those involving motorcycles.

Although a growing number of studies have examined the risk factors and trends associated with motorcycle crashes, as evidenced in Table 1, there is a notable research gap regarding motorcyclist injury severity in Cambodia, especially in relation to the distinct impacts of the rainy and dry seasons. To the best of the authors' knowledge, this specific aspect has not been extensively studied, highlighting a need for focused research in this area to understand how seasonal variations influence motorcycle crash outcomes in Cambodia.

## 3 Methodology

In reality, it is inherently difficult to account for all factors that impact crash injury severity, such as physiological differences between genders, psychological traits across age groups, the role of passengers, varying roadway conditions, vehicle characteristics, and external elements like time, weather, and environmental factors. These unobservable properties, known as unobserved features or unobserved heterogeneities, must be carefully accounted for to ensure unbiased, reliable, and consistent estimation results [69,78]. To tackle this issue, a statistical model that can handle potentially complex unobserved heterogeneity is necessary to produce the most accurate model estimates. In this study, we employ a random parameters logit model, which has gained popularity in recent empirical studies of injury severity. Additionally, we will consider the potential variability in the means and variances of random parameters to enhance the model's predictive capability [79].

Mannering and Bhat (2014) introduced a significant advancement in the methodological applications utilized for crash frequency data and crash injury severity analysis, paving the way for future methodological developments in crash research [78]. Among the key methodological challenges encountered with crash data, unobserved heterogeneity emerged as one of the fundamental issues, alongside endogeneity, risk compensation, and missing data. Addressing these challenges effectively, the random parameters multinomial logit model proved to be a valuable tool, particularly in handling the complexities associated with unobserved heterogeneity.

Starting with a fixed parameter multinomial logit model (MNL) function, the injury severity outcome k involved motorcyclist m is represented as follows:

(1) $$Y_{km}=\beta_{k}X_{km}+\varepsilon_{km}$$

where $Y_{km}$ denote the injury severity outcome k involved motorcyclist m. $X_{km}$ is a vector of exogenous crash-related variables (rider and environment characteristics, road condition, etc.) that affect injury severity k. And $\beta_{k}$ is a vector of estimable parameters, whereas $\varepsilon_{km}$ is an error item.

To eliminate the possible biased results from the unobserved heterogeneity, one or more parameter estimated in the vector $\beta_{k}$ are allowed to vary across crash observations. Regarding the possibility of unobserved heterogeneity in means and variances of random parameters, $\beta_{k}$ could be defined as a vector of estimable parameters that vary across crash observations, which could be defined as follows [69,[79], [80], [81], [82]]:

(2) $$\beta_{km}=\beta_{k}+\delta_{km}Z_{km}+\sigma_{km}\;\mathrm{EXP}\left(\omega_{km}W_{km}\right)v_{km}$$

where $\beta_{k}$ refers to the mean parameter estimate across all crash observations, $Z_{km}$ represents a vector of explanatory variables capturing heterogeneity in the mean that affect the mean of motorcycle injury severity level k, $\delta_{km}$ is the corresponding vector of estimable parameters, $W_{km}$ is a vector capturing heterogeneity in standard deviation $\sigma_{km}$ with corresponding parameter vector $\omega_{km}$, while $v_{km}$ are vectors of randomly distributed terms. The two vectors of attributes ($Z_{km}$ and $W_{km}$) allows the value of parameter $\beta_{km}$ vary across observations, while $Z_{km}$ and $W_{km}$ may contain potential heterogeneity attributes relating to vehicle, occupants, roadway, and environment, etc. If no significant variables are included in $W_{km}$, the model is only a heterogeneity in means one, while no significant variables are characterized in neither $Z_{km}$ or $W_{km}$ results in the random parameter model without any heterogeneity in means and variations.

Considering the crash-specific unobserved heterogeneity, we let the $\beta_{km}$ vector have a continues density function $Prob\left(\beta_{m}=\beta \right)=f\left(\beta |\varphi \right)$, where φ is a vector of parameters determining this function. So, the random parameter logit model results could be rewritten as [79,83]:

(3) $$P_{m}\left(k\right)=\int \frac{\mathrm{EXP}\left(\beta_{k}X_{km}\right)}{\sum_{\forall k}\;\mathrm{EXP}\left(\beta_{k}X_{km}\right)}\;f\left(\beta |\varphi \right)d\beta$$

where f(β|φ) is the density function which determine β. And φ is relating parameter vector describing the density function (mean and variance) of β.

Parameters for the random parameter logit model, following a normal distribution and accounting for heterogeneity in means and variances, were estimated via the simulated maximum likelihood method using 1000 Halton draws in NLOGIT 6.0 software. The selection of the normal distribution was based on its demonstrated suitability in crash severity estimation models compared to other density distributions such as uniform, triangular, and lognormal distributions, as shown in previous studies [2,84,85].

Furthermore, in order to provide a clear interpretation of the impacts of significant variables on injury severity outcomes, marginal effects were calculated across all crash observations. Since only indicator variables were utilized in this study, the marginal effect reflects the change in probability when the indicator changes from 0 to 1. Then, the corresponding average marginal effect value of the explanatory variables $X_{km}$ on n-th crash injury was calculated as follows [86,87]:

(4) $$\frac{\partial P_{m}\left(k\right)}{\partial X_{km}}=\bar{P_{m}\left(k\right)}\left[given\;X_{km}=1\right]-\bar{P_{m}\left(k\right)}[given\;X_{km}=0]$$

where $\bar{P_{m}\left(k\right)}$ is the average probability value across all crash observations.

## 4 Data description

**Fig. 1:**
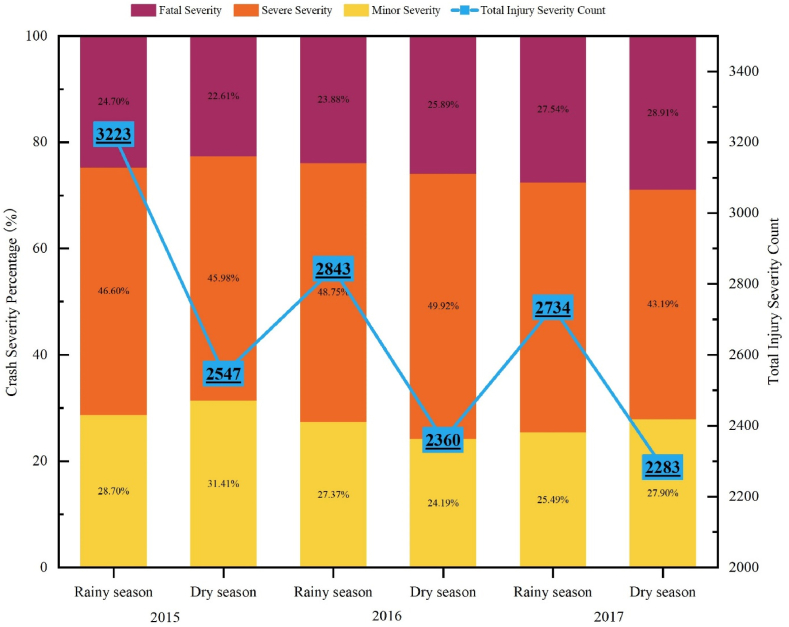
Seasonal motorcyclist injury severity distribution over the years.

This study utilized police-reported motorcycle crash data recorded by the Road Crash Victim and Information System (RCVIS) maintained by Cambodian National Road Safety Committee. The data includes motorcycle crashes that occurred between 2015 and 2017. After excluding cases with missing or unjustified records, a total of 15,990 recorded injuries were obtained. To account for Cambodia's weather patterns, the dataset was divided into two subsets: one during the rainy season (May 1st to November 30th) and the other during the dry season (December 1st to April 30th). The dependent variable adopted in this research is a categorical variable, referring to three levels of motorcycle injury severity: minor injury (MI), severe injury (SI), and fatal injury (FI). The year-wise rainy season and dry season injury severity percentage and frequency are shown in Fig. 1.

To validate the separation of rainy season and dry season casualties as distinct research groups in the study, a chi-square test was conducted for each year, as shown in Table 2. Results indicated that the null hypothesis – asserting that crash patterns in terms of injury severity levels are the same for both the rainy and dry seasons – could be confidently rejected for all the three years with confidence levels larger than 95 %. Moreover, the comparison of the number of casualties throughout the three-year period show that the number of total injuries generally tend to be declining but the number of seriously injured likely remains high and deteriorate over time, as the fatal injured casualties percentage increase year by year. This suggests that government and stakeholder initiatives to fight road traffic crash are effective in terms of containing the number of seriously injured but have had limited success in significantly reducing the total cases of injuries [7]. The steady rise in vehicle numbers, including both cars and motorcycles, each year due to population growth and increasing incomes, partly explains this trend [88].

**Table 2:** Motorcyclist injury observations distribution by severity level and Chi-square test.

	Minor		Severe		Fatal		Total	
	Rainy Season	Dry Season	Rainy Season	Dry Season	Rainy Season	Dry Season	Rainy Season	Dry Season
2015	925	800	1502	1171	796	576	3223	2547
Chi-square test	6.210(2) [>95 %]							
2016	778	571	1386	1178	679	611	2843	2360
Chi-square test	7.448(2) [>95 %]							
2017	697	637	1284	986	753	660	2734	2283
Chi-square test	6.996(2) [>95 %]							

Explanatory variables used in this research include the following four groups: crash characteristics, human factors, road features and temporal aspects.

(1) Crash characteristics: counterpart type, collision type, and crash size.
(2) Human factors: rider or pillion rider, age, gender, occupation, helmet use, and human error.
(3) Roadway features: road geometry, type of road, road surface condition, and whether the area is urban or rural.
(4) Temporal aspects: day of the week and time of day.

Basic statistical features of the above dependent and explanatory variables by season are shown in Table 3, respectively.

**Table 3:** Descriptive statistics of final motorcycle crash dataset.

Variables	2015 Rainy Season	2016 Rainy Season	2017 Rainy Season	2015 Dry Season	2016 Dry Season	2017 Dry Season
	Mean (Std)	Mean (Std)	Mean (Std)	Mean (Std)	Mean (Std)	Mean (Std)
**Crash Characteristics**						
Hit motorcycle (1 if a motorcycle was struck, 0 otherwise)	0.444 (0.247)	0.429 (0.245)	0.394 (0.239)	0.481 (0.25)	0.416 (0.243)	0.408 (0.241)
Hit pedestrian (1 if a pedestrian was struck, 0 otherwise)	0.267 (0.196)	0.294 (0.208)	0.305 (0.212)	0.224 (0.174)	0.289 (0.206)	0.295 (0.208)
Hit passenger vehicle (1 if a passenger vehicle was struck, 0 otherwise)	0.129 (0.112)	0.131 (0.114)	0.167 (0.139)	0.129 (0.112)	0.141 (0.121)	0.168 (0.14)
Head on collision (1 if it was head on collision, 0 otherwise)	0.364 (0.232)	0.33 (0.221)	0.334 (0.223)	0.379 (0.235)	0.362 (0.231)	0.371 (0.233)
Rear end collision (1 if it was rear end collision, 0 otherwise)	0.164 (0.137)	0.188 (0.153)	0.159 (0.134)	0.168 (0.14)	0.165 (0.138)	0.14 (0.121)
Right angle collision (1 if it was right angle collision, 0 otherwise)	0.261 (0.193)	0.272 (0.198)	0.317 (0.217)	0.249 (0.187)	0.265 (0.195)	0.325 (0.219)
Side swipe collision (1 if it was side swipe collision, 0 otherwise)	0.096 (0.087)	0.087 (0.08)	0.067 (0.063)	0.088 (0.08)	0.081 (0.074)	0.06 (0.056)
Fall alone (1 if it was fall-alone collision, 0 otherwise)	0.047 (0.044)	0.045 (0.043)	0.042 (0.04)	0.048 (0.045)	0.037 (0.035)	0.04 (0.038)
**Human Factors**						
Rider (1 if it was the rider victim, 0 otherwise)	0.668 (0.222)	0.672 (0.221)	0.677 (0.219)	0.661 (0.224)	0.67 (0.221)	0.671 (0.221)
Pillion rider (1 if it was the pillion rider victim, 0 otherwise)	0.332 (0.222)	0.328 (0.221)	0.323 (0.219)	0.338 (0.224)	0.33 (0.221)	0.329 (0.221)
Below 20 years old (1 if the victim is under 20, 0 otherwise)	0.216 (0.169)	0.232 (0.178)	0.23 (0.177)	0.226 (0.175)	0.23 (0.177)	0.249 (0.187)
20–29 years old (1 if the victim was aged 20 to 29, 0 otherwise)	0.420 (0.244)	0.379 (0.235)	0.393 (0.239)	0.419 (0.243)	0.399 (0.24)	0.387 (0.237)
30–39 years old (1 if the victim was aged 30 to 39, 0 otherwise)	0.177 (0.146)	0.196 (0.158)	0.178 (0.146)	0.166 (0.138)	0.179 (0.147)	0.169 (0.14)
40–49 years old (1 if the victim was aged 40 to 49, 0 otherwise)	0.087 (0.080)	0.09 (0.082)	0.092 (0.083)	0.086 (0.078)	0.091 (0.083)	0.089 (0.082)
Above 50 years old (1 if the victim was 50, 0 otherwise)	0.099 (0.089)	0.103 (0.092)	0.108 (0.096)	0.104 (0.093)	0.102 (0.092)	0.106 (0.094)
Male (1 if it was the male victim, 0 otherwise)	0.795 (0.163)	0.795 (0.163)	0.807 (0.156)	0.792 (0.165)	0.792 (0.165)	0.795 (0.163)
Female (1 if was the female victim, 0 otherwise)	0.205 (0.163)	0.205 (0.163)	0.193 (0.156)	0.208 (0.165)	0.207 (0.166)	0.205 (0.163)
Wearing helmet (1 if the victim wore helmet, 0 otherwise)	0.137 (0.118)	0.246 (0.186)	0.213 (0.168)	0.127 (0.111)	0.231 (0.178)	0.177 (0.146)
Speeding (1 if the victim was speeding, 0 otherwise)	0.326 (0.220)	0.289 (0.205)	0.279 (0.201)	0.328 (0.221)	0.308 (0.213)	0.257 (0.191)
Not respect right of way (1 if the victim failed to yield the right of way, 0 otherwise)	0.099 (0.089)	0.08 (0.074)	0.147 (0.126)	0.116 (0.102)	0.097 (0.876)	0.125 (0.109)
Driving against flow of traffic (1 if the victim was traveling against the traffic flow, 0 otherwise)	0.057 (0.054)	0.093 (0.084)	0.116 (0.102)	0.073 (0.068)	0.116 (0.123)	0.16 (0.135)
Dangerous overtaking (1 if the victim was overtaking dangerously, 0 otherwise)	0.129 (0.112)	0.134 (0.116)	0.127 (0.111)	0.13 (0.113)	0.116 (0.102)	0.134 (0.116)
Alcohol abuse (1 if the victim was impaired due to alcohol abuse, 0 otherwise)	0.165 (0.138)	0.124 (0.109)	0.12 (0.105)	0.173 (0.143)	0.128 (0.111)	0.103 (0.092)
**Roadway Features**						
National Road (1 if the crash occurred on a national road, 0 otherwise)	0.597 (0.241)	0.612 (0.238)	0.604 (0.239)	0.573 (0.225)	0.583 (0.243)	0.617 (0.236)
Provincial Road (1 if the crash occurred on a provincial road, 0 otherwise)	0.197 (0.158)	0.147 (0.125)	0.15 (0.128)	0.187 (0.152)	0.169 (0.14)	0.164 (0.137)
Major Road (1 if the crash occurred on a major road, 0 otherwise)	0.022 (0.021)	0.025 (0.025)	0.038 (0.036)	0.024 (0.023)	0.027 (0.027)	0.036 (0.035)
Minor Road (1 if the crash occurred on a minor road, 0 otherwise)	0.036 (0.034)	0.053 (0.05)	0.068 (0.063)	0.037 (0.035)	0.059 (0.056)	0.064 (0.06)
Local Road (1 if the crash occurred on a local road, 0 otherwise)	0.141 (0.121)	0.128 (0.111)	0.113 (0.1)	0.157 (0.132)	0.119 (0.105)	0.102 (0.091)
Paved (1 if the crash occurred on a paved road, 0 otherwise)	0.848 (0.129)	0.849 (0.128)	0.801 (0.159)	0.814 (0.152)	0.827 (0.143)	0.817 (0.15)
Cemented (1 if the crash occurred on a cemented road, 0 otherwise)	0.032 (0.031)	0.04 (0.039)	0.08 (0.074)	0.029 (0.028)	0.039 (0.037)	0.08 (0.074)
Urban (1 if the crash occurred in urban area, 0 otherwise)	0.411 (0.242)	0.401 (0.24)	0.422 (0.244)	0.359 (0.23)	0.417 (0.243)	0.423 (0.244)
Rural (1 if the crash occurred in rural area, 0 otherwise)	0.096 (0.087)	0.073 (0.067)	0.071 (0.066)	0.125 (0.109)	0.082 (0.076)	0.063 (0.059)
**Temporal Aspects**						
Weekday (1 if the crash occurred on weekday, 0 otherwise)	0.671 (0.221)	0.65 (0.228)	0.658 (0.225)	0.704 (0.208)	0.658 (0.225)	0.685 (0.216)
Weekend (1 if the crash occurred on weekend, 0 otherwise)	0.329 (0.221)	0.35 (0.228)	0.342 (0.225)	0.296 (0.208)	0.342 (0.225)	0.315 (0.216)

## 5 Transferability and temporal stability tests

Recent research has demonstrated that factors influencing crash injury severity change over time [81,82,86,[89], [90], [91], [92], [93], [94], [95]]. Regarding temporal stability testing, the global test and the pairwise comparison test are two popular approaches. Hou et al. (2022) found that the pairwise comparison method offers more detailed information compared to the global test. It was also noted that the temporal instability test could be considered a special case of transferability test [95]. Therefore, in this paper, both transferability and temporal stability use the pairwise likelihood ratio testing method to test.

These tests were conducted using likelihood ratio tests as outlined below [79]:

(5) $$\chi_{m2,m1}^{2}=-2\left[LL\left(\beta_{m2m1}\right)-LL\left(\beta_{m1}\right)\right]$$

where m denotes the rainy season or dry season for each year in the transferability test. $LL\left(\beta_{m2m1}\right)$ represents the log-likelihood at convergence for the model estimated using converged parameters from m1 season (either rainy or dry) applied to the data from m2 season (either rainy or dry, and m2≠m1) in the same year. $LL\left(\beta_{m1}\right)$ is the log-likelihood at convergence of the m1 season model using m1 season data with parameters.

(5) $$\chi_{t2,t1}^{2}=-2\left[LL\left(\beta_{t2t1}\right)-LL\left(\beta_{t1}\right)\right]$$

where t denotes each year of the crash (either 2015, 2016, or 2017) in the two seasonal temporal instability test. $LL\left(\beta_{t2t1}\right)$ is the log-likelihood at convergence of the model estimated using converged parameters from t1 period (either 2015, 2016, or 2017 with rainy season, or dry season model) on data in t2 period (either 2015, 2016, or 2017 with rainy season, or dry season model, and t2≠t1). $LL\left(\beta_{t1}\right)$ represents the log-likelihood of the model from t1 period, estimating using the data and parameters from the same period. The tests were also reversed, with the period t1 swapped for t2 and vice versa.

For the transferability test, the null hypothesis assumes that the parameters are equal between the rainy and dry season. The transferability tests results for each pair of periods are presented in Table 4. The test results strongly rejected the null hypothesis, indicating significant differences in parameters between rainy and dry season data for each year. The confidence levels for rejecting the null hypothesis were found to be reasonably high.

**Table 4:** Transferability test results between rainy season and dry season crashes for each year.

Years	m2	Dry season	Rainy season
	m1	Rainy season	Dry season
2015		128.467 [67] (>99.99 %)	95.413 [67] (>99.99 %)
2016		231.086 [21] (>99.99 %)	143.259 [68] (>99.99 %)
2017		140.036 [24] (>99.99 %)	258.137 [23] (>99.99 %)

The temporal stability test results for rainy season and dry season data between each year are shown in Table 5, Table 6, respectively. In all cases, the results indicate that both the model specifications and estimated parameters show temporal and seasonal instability across the majority of period combinations in a confidence level of 99.99 %. Further details on individual explanatory variable findings will be provided in the discussion section below.

**Table 5:** Temporal stability test results of rainy season crashes.

t1	t2		
	2015	2016	2017
2015	–	140.538 [67] (>99.99 %)	135.176 [67] (>99.99 %)
2016	150.671 [21] (>99.99 %)	–	128.140 [21] (>99.99 %)
2017	271.757 [24] (>99.99 %)	167.186 [24] (>99.99 %)	–

**Table 6:** Temporal stability test results of dry season crashes.

t1	t2		
	2015	2016	2017
2015	–	153.176 [67] (>99.99 %)	297.562 [67] (>99.99 %)
2016	110.456 [68] (>99.99 %)	–	230.146 [68] (>99.99 %)
2017	95.146 [23] (>99.99 %)	115.063 [23] (>99.99 %)	–

The results emphasize the significance of assessing the transferability and temporal instability of factors impacting motorcyclist injuries, especially when comparing different seasons. Additionally, these findings align to some extent with the results reported in Ref. [89].

## 6 Results and discussion

In this section, we investigate the significant variables (at 90 % significant level) and how they influence the likelihood of motorcyclist injuries throughout rainy seasons and dry seasons spanning 2015 to 2017. Table 7 provides respective estimated outcomes of motorcycle crashes across these designated seasons within the specified timeframe. Notably, all of the random parameter's coefficient revealed in the table are statistically significant at 90 % confidence level. Throughout all the year-wise rainy and dry season models, heterogeneity in the means of the random parameters was observed. However, their heterogeneity in the variances was evident across models except for the 2015 rainy season. Furthermore, Table 8 presents a comprehensive summary detailing the average marginal effects of motorcyclist injury severity (including minor, severe), and fatal determinants, during both the rainy seasons and dry seasons for each subsequent year.

**Table 7:** Parameter estimation results for motorcycle crashes in both rainy and dry season (z-score in parentheses).

Variable	2015		2016		2017	
	Rainy Season	Dry Season	Rainy Season	Dry Season	Rainy Season	Dry Season
**Constant [MI]**		−0.066 (−2.108)				
**Constant [SI]**	−1.106 (−4.804)		−1.050 (−4.147)	−0.955 (−4.019)	−0.985 (−3.262)	−1.022 (−3.983)
**Constant [FI]**	−3.259 (−9.132)	−2.065 (−5.269)	−2.372 (−5.554)	−2.328 (−6.665)	−4.421 (−6.412)	−3.410 (−8.339)
**Crash Characteristics**						
[SI] Hit motorcycle (1 if a motorcycle was struck, 0 otherwise)	0.326 (1.878)		0.567 (3.030)			0.466 (2.400)
[FI] Hit motorcycle 1 if a motorcycle was struck, 0 otherwise)		−0.625 (−2.408)	−0.652 (−2.238)	1.243 (6.984)	−0.703 (−2.072)	
[FI] *Standard deviation of “Hit motorcycle”*						2.943 (2.617)
[SI] Hit pedestrian (1 if a pedestrian was struck, 0 otherwise)	0.935 (5.231)	0.625 (3.722)	1.257 (6.471)			0.985 (5.064)
[FI] Hit pedestrian (1 if a pedestrian was struck, 0 otherwise)	1.237 (5.233)			1.236 (5.440)	0.698 (2.277)	
[FI] *Standard deviation of “Hit pedestrian”*					2.370 (2.774)	
[MI] Hit passenger vehicle (1 if a passenger vehicle was struck, 0 otherwise)		1.247 (3.457)				
[SI] Hit passenger vehicle (1 if a passenger vehicle was struck, 0 otherwise)			1.168 (4.749)		0.407 (1.748)	1.314 (5.566)
[FI] Hit passenger vehicle (1 if a passenger vehicle was struck, 0 otherwise)	1.895 (6.814)			1.804 (6.617)	1.489 (4.516)	
[FI] *Standard deviation of “Hit passenger vehicle”*						3.114 (2.103)
[MI] Head on collision (1 if it was head on collision, 0 otherwise)		−0.257 (2.578)				
[SI] Head on collision (1 if it was head on collision, 0 otherwise)	0.481 (3.372)				0.930 (5.344)	0.285 (2.225)
[FI] Head on collision (1 if it was head on collision, 0 otherwise)			0.794 (3.679)	0.869 (3.631)	1.386 (5.118)	0.885 (4.906)
[FI] Right angle collision (1 if it was right angle collision, 0 otherwise)		−0.926 (−3.777)				
[SI] Side swipe collision (1 if it was side swipe collision, 0 otherwise)	−0.337 (−1.735)					−0.538 (−2.658)
[FI] Side swipe collision (1 if it was side swipe collision, 0 otherwise)		−2.165 (−2.653)	−0.911 (−2.655)	−0.894 (−3.095)	1.376 (−2.650)	−0.820 (−2.989)
[FI] *Standard deviation of “*Side swipe collision*”*		2.832 (2.395)				
[SI] Fall alone (1 if it was fall-alone collision, 0 otherwise)			0.705 (2.286)			
[FI] Fall alone (1 if it was fall-alone collision, 0 otherwise)	1.268 (3.785)	1.108 (3.000)		1.954 (4.276)	1.541 (3.007)	0.948 (2.632)
**Human Factors**						
[MI] Rider (1 if it was the rider victim, 0 otherwise)		−0.238 (4.018)				
[SI] Rider (1 if it was the rider victim, 0 otherwise)	0.696 (6.180)		0.254 (2.064)	0.443 (4.011)		
[FI] Rider (1 if it was the rider victim, 0 otherwise)	1.262 (7.464)	1.216 (5.166)	0.813 (3.046)	0.963 (5.521)	1.446 (4.687)	0.771 (4.472)
[SI] Male (1 if it was the male victim, 0 otherwise)						0.201 (1.651)
[FI] Male (1 if it was the male victim, 0 otherwise)						0.603 (3.303)
[FI] 30–39 years old (1 if the victim was aged 30 to 39, 0 otherwise)			0.841 (2.878)			0.438 (2.100)
[MI] 40–49 years old (1 if the victim was aged 40 to 49, 0 otherwise)		−0.307 (3.176)				
[SI] 40–49 years old (1 if the victim was aged 40 to 49, 0 otherwise)			0.567 (2.784)	0.319 (1.737)		0.428 (2.109)
[FI] 40–49 years old (1 if the victim was aged 40 to 49, 0 otherwise)	0.606 (2.631)	0.673 (2.699)		0.733 (3.158)	0.603 (1.790)	0.903 (3.537)
[SI] Above 50 years old (1 if the victim was 50, 0 otherwise)						0.451 (2.312)
[FI] Above 50 years old (1 if the victim was 50, 0 otherwise)	1.474 (6.547)	1.049 (4.278)	0.860 (3.331)	1.098 (4.774)		
[SI] Helmet (1 if the victim wore helmet, 0 otherwise)				−0.270 (−2.280)	−0.253 (−1.704)	
[FI] Helmet (1 if the victim wore helmet, 0 otherwise)	−0.978 (−4.730)	−0.564 (−2.486)	−0.519 (−2.727)	−0.556 (−2.278)	−0.765 (−3.343)	−0.612 (−3.564)
[SI] Speeding (1 if the victim was speeding, 0 otherwise)	0.218 (1.810)					
[FI] Speeding (1 if the victim was speeding, 0 otherwise)	0.451 (2.364)	0.362 (1.939)	1.916 (2.732)		0.308 (1.662)	0.404 (1.903)
[FI] *Standard deviation of “Speeding”*	1.672 (2.303)		2.099 (2.949)		2.310 (3.259)	
[FI] Overtaking (1 if the victim was overtaking dangerously, 0 otherwise)					0.862 (2.856)	
[SI] Against flow (1 if the victim was traveling against the traffic flow, 0 otherwise)			0.428 (2.550)			
[FI] Against flow (1 if the victim was traveling against the traffic flow, 0 otherwise)						0.588 (2.352)
[SI] Alcohol (1 if the victim was impaired due to alcohol abuse, 0 otherwise)	−0.275 (−1.755)					
[FI] Alcohol (1 if the victim was impaired due to alcohol abuse, 0 otherwise)	1.236 (6.171)	0.762 (3.098)			0.947 (3.995)	0.910 (4.025)
[FI] *Standard deviation of “Alcohol”*					2.237 (2.191)	
**Roadway Features**						
[SI] National road (1 if the crash occurred on a national road, 0 otherwise)				0.296 (2.998)		
[FI] National road (1 if the crash occurred on a national road, 0 otherwise)		0.437 (4.734)	0.628 (2.765)	0.626 (3.316)	0.673 (2.847)	0.530 (3.557)
[FI] Major road (1 if the crash occurred on a major road, 0 otherwise)	1.274 (2.618)			1.199 (3.190)		
[SI] Urban (1 if the crash occurred in urban area, 0 otherwise)			−0.213 (−1.976)			
[FI] Urban (1 if the crash occurred in urban area, 0 otherwise)	−0.354 (−2.510)			−0.852 (−6.015)	−0.619 (−3.839)	
[FI] *Standard deviation of “Urban”*					2.054 (3.717)	4.916 (2.473)
**Temporal Aspects**						
[SI] Weekend (1 if the crash occurred on weekend, 0 otherwise)					0.182 (1.790)	
[FI] Weekend (1 if the crash occurred on weekend, 0 otherwise)	0.250 (1.732)				0.461 (2.363)	
**Heterogeneity in the means of the random parameter**						
[FI] Side swipe collision (1 if it was side swipe collision, 0 otherwise): Hit pedestrian (1 if a pedestrian was struck, 0 otherwise)	−2.443 (−3.066)					
[FI] Alcohol (1 if the victim was impaired due to alcohol abuse, 0 otherwise): Hit pedestrian (1 if a pedestrian was struck, 0 otherwise)		−1.942 (−2.617)				
[FI] National road (1 if the crash occurred on a national road, 0 otherwise): Hit passenger vehicle (1 if a passenger vehicle was struck, 0 otherwise)		1.985 (2.867)				
[FI] Urban (1 if the crash occurred in urban area, 0 otherwise): Hit pedestrian (1 if a pedestrian was struck, 0 otherwise)		−1.931 (−2.766)				
[FI] Helmet (1 if the victim wore helmet, 0 otherwise): Right angle collision (1 if it was right angle collision, 0 otherwise)				−1.482 (−2.641)		
[FI] Helmet (1 if the victim wore helmet, 0 otherwise): Hit pedestrian (1 if a pedestrian was struck, 0 otherwise)				1.617 (2.924)		
[FI] National road (1 if the crash occurred on a national road, 0 otherwise): Head on collision (1 if it was head on collision, 0 otherwise)				−1.094 (−2.376)		
[FI] National road (1 if the crash occurred on a national road, 0 otherwise): Fall alone (1 if it was fall-alone collision, 0 otherwise)				−2.691 (2.163)		
[FI] Speeding (1 if the victim was speeding, 0 otherwise): Male (1 if it was the male victim, 0 otherwise e)					1.729 (2.263)	
[FI] Alcohol (1 if the victim was impaired due to alcohol abuse, 0 otherwise): Male (1 if it was the male victim, 0 otherwise)					2.759 (2.250)	
[FI] Hit pedestrian (1 if a pedestrian was struck, 0 otherwise): Weekend (1 if the crash occurred on weekend, 0 otherwise)						4.762 (2.036)
[FI] Hit motorcycle (1 if a motorcycle was struck, 0 otherwise): Speeding (1 if the victim was speeding, 0 otherwise)						1.512 (2.048)
**Heterogeneity in the variances of the random parameter**						
[FI] Urban (1 if the crash occurred in urban area, 0 otherwise): Side swipe collision (1 if it was side swipe collision, 0 otherwise)		1.017 (1.904)				
[FI] Head on collision (1 if it was head on collision, 0 otherwise): Rider (1 if it was the rider victim, 0 otherwise)			−0.977 (−2.264)			
[FI] National road (1 if the crash occurred on a national road, 0 otherwise): Rider (1 if it was the rider victim, 0 otherwise)				−0.582 (−1.705)		
[FI] Hit pedestrian (1 if a pedestrian was struck, 0 otherwise): Rider (1 if it was the rider victim, 0 otherwise)					0.620 (2.000)	
[FI] Speeding (1 if the victim was speeding, 0 otherwise): Rider (1 if it was the rider victim, 0 otherwise)					1.336 (2.088)	
[FI] Hit motorcycle (1 if a motorcycle was struck, 0 otherwise): Rider (1 if it was the rider victim, 0 otherwise)						2.238 (1.799)
**Model statistics**						
Number of parameters (K)	23	23	21	24	29	28
Number of observations (N)	3223	2547	2843	2360	2734	2283
Log-likelihood at zero	−3329.520	−2841.722	−3055.952	−2518.103	−2913.582	−2346.050
Log-likelihood at convergence	−2587.037	−2298.953	−2501.506	−2027.073	−2444.017	−1893.026
AIC	5220.074	4643.906	5045.012	4102.146	4946.034	3842.052
McFadden ρ2	0.223	0.191	0.201	0.195	0.183	0.209

**Table 8:** Marginal effects of determinants for motorcyclist crashes in rainy and dry season (marginal effects of dry season model in parentheses).

Variable	MI			SI			FI		
	2015	2016	2017	2015	2016	2017	2015	2016	2017
**Crash Characteristics**									
Hit motorcycle (1 if a motorcycle was struck, 0 otherwise)	−0.0107 (−0.0048)	−0.0181 (−0.0203)	−0.0122 (−0.0130)	0.0213 (0.0199)	0.0354 (0.0331)	0.0351 (0.0280)	−0.0106 (−0.0151)	−0.0173 (−0.0128)	−0.0229 (−0.0150)
Hit pedestrian (1 if a pedestrian was struck, 0 otherwise)	−0.0192 (−0.0110)	−0.0227 (−0.0233)	−0.0159 (−0.0185)	0.0073 (0.0042)	0.0163 (0.0172)	0.0091 (0.0184)	0.0119 (0.0068)	0.0064 (0.0061)	0.0068 (0.0001)
Hit passenger vehicle (1 if a passenger vehicle was struck, 0 otherwise)	−0.0102 (−0.0069)	−0.0102 (−0.0109)	−0.0085 (−0.0112)	−0.0028 (−0.0023)	−0.0014 (−0.0006)	−0.0055 (0.0025)	0.0129 (0.0092)	0.0116 (0.0115)	0.0141 (0.0088)
Head on collision (1 if it was head on collision, 0 otherwise)	−0.0166 (−0.0153)	−0.0149 (−0.0177)	−0.0249 (−0.0108)	0.0037 (0.0128)	0.0061 (0.0056)	0.0107 (−0.0069)	0.0129 (−0.0025)	0.0088 (0.0121)	0.0142 (0.0177)
Right angle collision (1 if it was right angle collision, 0 otherwise)	- (0.0068)	0.0025 (−0.0034)	- (−)	- (0.0018)	0.0069 (0.0088)	- (−)	- (−0.0086)	−0.0094 (−0.0054)	- (−)
Side swipe collision (1 if it was side swipe collision, 0 otherwise)	0.0034 (0.0037)	0.0021 (0.0025)	0.0001 (0.0040)	−0.0019 (−0.0025)	0.0013 (0.0007)	0.0045 (−0.0021)	−0.0015 (−0.0012)	−0.0034 (−0.0031)	−0.0046 (−0.0019)
Fall alone (1 if it was fall-alone collision, 0 otherwise)	−0.0031 (−0.0023)	−0.0024 (−0.0029)	−0.0018 (−0.0019)	−0.0001 (−0.0004)	0.0007 (−0.0002)	−0.0014 (−0.0005)	0.0032 (0.0027)	0.0017 (0.0031)	0.0031 (0.0025)
**Human Factors**									
Rider (1 if it was the rider victim, 0 otherwise)	−0.0242 (−0.0416)	−0.0284 (−0.0354)	−0.0332 (−0.0456)	0.0065 (0.0065)	0.0095 (0.0032)	0.0092 (0.0142)	0.0177 (0.0351)	0.0189 (0.0322)	0.0240 (0.0314)
Male (1 if it was the male victim, 0 otherwise)			- (−0.0357)			- (−0.0084)			- (−0.0273)
30–39 years old (1 if the victim was aged 30 to 39, 0 otherwise)	−0.0037 (−0.0033)	−0.0069 (−0.0023)	- (−0.0039)	0.0025 (0.0019)	−0.0008 (0.0014)	- (0.0007)	0.0012 (0.0014)	0.0077 (0.0009)	- (0.0033)
40–49 years old (1 if the victim was aged 40 to 49, 0 otherwise)	−0.0029 (−0.0026)	−0.0038 (−0.0027)	−0.0023 (−0.0035)	0.0006 (0.0003)	0.0017 (−0.0010)	0.0003 (−0.0009)	0.0023 (0.0023)	0.0022 (0.0037)	0.0020 (0.0044)
Above 50 years old (1 if the victim was 50, 0 otherwise)	−0.0058 (−0.0035)	−0.0047 (−0.0051)	−0.0032 (−0.0047)	−0.0017 (−0.0024)	0.0018 (0.0015)	−0.0014 (−0.0027)	0.0075 (0.0059)	0.0028 (0.0036)	0.0045 (0.0074)
Helmet (1 if the victim wore helmet, 0 otherwise)	0.0065 (0.0036)	0.0019 (0.0070)	0.0053 (0.0041)	−0.0007 (−0.0008)	0.0061 (0.0012)	0.0006 (0.0033)	−0.0059 (−0.0028)	−0.0080 (−0.0082)	−0.0059 (−0.0074)
Speeding (1 if the victim was speeding, 0 otherwise)	−0.0147 (−0.0022)	−0.0126 (−)	−0.0037 (−0.0019)	0.0024 (−0.0048)	0.0053 (−)	−0.0075 (−0.0124)	0.0123 (0.0071)	0.0073 (−)	0.0112 (0.0142)
Overtaking (1 if the victim was overtaking dangerously, 0 otherwise)	−0.0039 (−)	−0.0036 (−)	−0.0003	−0.0003 (−)	−0.0043 (−)	−0.0089	0.0042 (−)	0.0079 (−)	0.0092 (0.0020)
Against flow (1 if the victim was traveling against the traffic flow, 0 otherwise)	- (−)	0.0011 (−)	- (−0.0038)	- (−)	−0.0047 (−)	- (0.0018)	- (−)	0.0035 (−)	- (0.0020)
Alcohol (1 if the victim was impaired due to alcohol abuse, 0 otherwise)	−0.0079 (−0.0029)	- (−)	−0.0023 (−0.0040)	−0.0078 (−0.0032)	- (−)	−0.0027 (−0.0035)	0.0158 (0.0060)	- (−)	0.0050 (0.0075)
**Roadway Features**									
National road (1 if the crash occurred on a national road, 0 otherwise)	−0.0068 (−0.0163)	−0.0048 (−0.0180)	−0.0068 (−0.0048)	−0.0154 (−0.0088)	−0.0090 (−0.0034)	−0.0082 (−0.0220)	0.0222 (0.0251)	0.0138 (0.0214)	0.0150 (0.0269)
Major road (1 if the crash occurred on a major road, 0 otherwise)	−0.0010 (−)	- (−0.0012)	- (−)	−0.0004 (−)	- (−0.0007)	- (−)	0.0015 (−)	- (0.0020)	- (−)
Urban (1 if the crash occurred in urban area, 0 otherwise)	0.0053 (−)	0.0123 (0.0173)	0.0046 (0.0133)	0.0024 (−)	−0.0015 (−0.0018)	−0.0014 (−0.0115)	−0.0077 (−)	−0.0108 (−0.0155)	−0.0032 (−0.0218)
**Temporal Aspects**									
Weekend (1 if the crash occurred on weekend, 0 otherwise)	−0.0031 (−)	- (−)	−0.0041 (−)	−0.0017 (−)	- (−)	−0.0031 (−)	0.0048 (−)	- (−)	0.0072 (−)

∗MI = Minor Injury; SI=Severe Injury; FI=Fatal Injury.

The next sections are structured as: Section 6.1, 6.2, 6.3, 6.4 discuss the average marginal effect values of statistically significant parameters related to crash characteristics, human factors, roadway features, and temporal factors, respectively. Section 6.5 presents the results and discussions about the random parameter results and heterogeneity in means and variances.

The estimation results of the above models highlight varying impacts of significant factors, characterized by random effects and their heterogeneity in the means and variances. These variations are observable over different time frames, including over three-year periods and between the rainy and dry seasons. The following section provides a detailed discussion of these modeling results. Additionally, we address the temporal instability of these determinants during both seasons from 2015 to 2017 to contribute to further enhancement of motorcyclist safety.

### 6.1 Crash-related characteristics

In all rainy and dry season models, motorcycle-to-motorcycle crashes were statistically significant, showing stable average marginal effects that raised the probability of severe injuries and lowered the probability of fatal injuries (Table 8). One potential explanation for this phenomenon is that when two motorcycles collide, the crash impact might be comparatively lower than in collisions involving motorcycles and larger vehicles, as evidenced by existing research [2,96,97]. Given the relatively higher marginal effects of severe and fatal injuries associated with the “hit motorcycle” crash variable compared to other types of crashes, it is crucial to allocate additional attention and efforts to reduce hit motorcycle crashes involving motorcycle users.

Throughout 2015–2017, the variable representing motorcycle casualties involved in hitting pedestrian crashes exhibited statistical significance in both rainy and dry season models, consistently increasing the likelihood of severe and fatal injuries (as depicted in Table 8). One plausible explanation for this observation is that riders tend to exercise less caution when colliding with a pedestrian, potentially leading to more severe outcomes. Additionally, it is worth noting that the variance of the random parameter in 2017 rainy season crashes also demonstrated significance, indicating potential variability in the impact of this particular factor during that specific period.

The variable representing casualties involved in hitting passenger vehicle crashes displayed statistical significance in all rainy and dry season models, as it consistently heightens the probability of fatal injury (as indicated in Table 8). Notably, the “hitting passenger cars” marginal effect was observed more severe in rainy seasons compared to dry seasons. This outcome is consistent with our intuitive understanding, as collision mechanisms involving motorcycles and Passenger vehicles are generally more likely to result in more severe crashes. As a result, the severity of the injuries sustained in such accidents tends to be greater. Moreover, these findings find support in various prior studies [29,[97], [98], [99], [100]]. These studies have also highlighted the increased risk of severe injuries when motorcycles collide with passenger vehicles. Additionally, it is pertinent to mention that the variance of the random parameter in 2016 and 2017 dry seasons exhibits significance, signifying potential variability in the impact of this factor during that specific period.

As evident from the data presented in Table 8, head-on crashes demonstrated statistical significance with regards to severe and fatal severity in all rainy and dry season models. It heightened the risk of severe and fatal injuries while simultaneously reducing the likelihood of minor injuries. This consistent marginal effect was independent of the time-of-year. These findings align with logical expectations and are approved by substantial literature [37,92]. Given the relatively high marginal effect of fatal injuries associated with the head-on crash variable, it becomes imperative to place extra emphasis on minimizing the incidence of motorcycle head-on crashes. All of this evidence emphasizes the importance of implementing measures to address this specific type of crash, which poses a significant risk to the safety of motorcyclists.

In analyzing the indicator for side swipe collisions, it was observed that it had a statistically significant influence in reducing the risk of fatal injuries in both rainy season and dry season models throughout the three-year period. It was also found that the same indicator significantly decreases the severe injury severity in 2015 rainy season, 2015 dry season, and 2017 dry season. These results highlight the complex nature of side swipe collisions and their diverse effects on different injury severities. The findings underscore the importance of understanding the specific circumstances and dynamics surrounding side swipe collisions to effectively address their impact on motorcycle safety. Further research and interventions are warranted to develop targeted measures that mitigate severe injuries while also reducing the potential for fatal outcomes in these types of collisions.

In the analysis of fall-alone crashes, the indicator exhibited statistical significance in the models for the years 2015, and 2016, with the marginal effects consistently increasing the likelihood of severe injury and fatal injury in both rainy season and dry season scenarios. However, it is worth noting that in the specific case of 2017 rainy season and dry season crashes, the indicator was only significantly increase the probability of fatal injury. These results highlight the varying effects of fall alone crashes on different injury severities across various time periods. The findings emphasize the importance of comprehensively studying the contributing factors and circumstances surrounding fall-alone crashes to better understand the potential risks they pose to motorcyclists. Further investigation into the underlying causes of these crashes is crucial for the development of targeted interventions and safety measures aimed at mitigating both severe and fatal injuries associated with fall-alone incidents.

In relation to the various crash types examined in this study, we observed both seasonal non-transferability and temporal instability between the six models. This phenomenon could be due to several significant sources of variations, which could not be fully accounted for due to certain limitations in the current research. One important source of variation lies in the crash determination concerning the vehicle collision path. An example provided by Ref. [24] illustrates the challenge of differentiating between side swipe and right-angle crashes based on the crash determination provided by officers, as both types involve same-direction collisions between two vehicles moving with the same direction. Other factors also exert varying influences on the injury severity level. These factors encompass elements such as controlled/uncontrolled intersections or junctions, the direction of another vehicle, roadway markings, right-of-way, and shoulder width [24 101,102]. Additionally, at-fault or not-at-fault riders also emerge as a potential source of variation [103]. For instance, if not-at-fault riders experience a rear-end collision or a sideswipe from another vehicle, they are more likely to be ejected, leading to a higher likelihood of sustaining severe injuries [24]. It is crucial to acknowledge that this aspect also represents a limitation of our current research. To address these limitations and enhance the comprehensiveness of future studies, it would be valuable to gather a more comprehensive dataset, encompassing a wider range of factors and crash circumstances. Taking these measures will significantly contribute to the progress of motorcycle safety research and aid in the development of focused interventions. These efforts are key to decreasing injuries and fatalities on the roads, ultimately enhancing overall road safety.

### 6.2 Human-related characteristics

Compared to pillion riders, the average marginal effects of motorcycle riders significantly increase the probability of severe and fatal injury in all year models (as shown in Table 8). However, it is essential to note that the marginal effects scale exhibited fluctuations across all the six models. This observation aligns with logical expectations and previous research [81]. The findings indicate that being the rider in a crash may increase the likelihood of sustaining more severe injuries compared to the pillion rider [[103], [104]]. These results contribute to our understanding of the risks faced by motorcycle riders in comparison to pillion riders during crashes. The study highlights the importance of considering the role of the rider in injury outcomes and underscores the need for targeted measures to enhance rider safety and mitigate injury severity. Further investigation into the factors contributing to the observed fluctuations in marginal effects across different models and time periods would be valuable for the development of effective strategies to reduce motorcycle crash injuries.

Statistical insignificance of the male indicator was observed only in the 2017 dry season model, indicating non-transferability among seasonal and yearly models. This implies that dry season motorcycle crashes for each year should be modeled separately. However, in the 2017 dry season model, the male indicator exhibited statistical significance with increasing probability of more severe injuries. Previous studies provide support for this result [34], whereas other studies present contradictory results [37]. This discrepancy may potentially be attributed to differences in the attitudes and behaviors between male and female motorcyclists in motorcycle safety studies [105]. A plausible reason for these differences might be that males are more likely to exhibit extroverted personality traits compared to females. Extroverted individuals are often more likely to take higher risks in search of excitement and thrills. This inclination can make extroverted motorcyclists more vulnerable to accidents compared to their introverted counterparts [26]. Additionally, males tend to be more vulnerable to aggressive driving, substance abuse, and risk-taking behaviors than females [106,107].

The results generally indicate that elderly riders are at a greater risk of sustaining severe injuries than younger riders. The differing levels of experience among motorcyclists across age groups might be a possible explanation for this disparity. Riders over 30, who tend to be more experienced, may become overconfident when faced with uncontrolled conditions. As a result, once a crash occurs, injuries are more severe due to a lack of sufficient risk compensations to neutralize the heightened risk of injury in such circumstances. In essence, the superior motorcycling skills possessed by riders over 30 years old may be offset by inadequate risk compensatory behaviors when dealing with crashes in such circumstances. This finding underscores the importance of considering age and experience levels when assessing motorcycle crash outcomes, as it provides valuable insights into potential risk-taking behaviors and safety implications for different age groups of motorcyclists. Further research could delve deeper into the factors contributing to these age-related differences in injury severity to develop targeted interventions and safety measures aimed at reducing injuries and enhancing road safety for motorcyclists of all age groups.

In all rainy and dry season models, the helmet-wearing variable was found to be significant, consistently decreasing the probability of fatal injury (as shown in Table 8). However, it's worth noting that helmet use did not significantly influence severe injury severity in the 2016 rainy season and 2017 dry season models. These findings demonstrate the effectiveness of helmet use in reducing injury severity [108]. This observation highlights the critical importance of helmet usage for both riders and pillion passengers in minimizing injury severity.

Speeding-related crashes showed considerable instability across different seasons and in various yearly models. In the rainy season crash models, the speeding variable showed significant parameters on fatal injury severity during all the time periods, but it only significantly impacted severe injuries in 2015 and 2016 (as indicated in Table 8). However, in the dry seasons of 2015 and 2017, speeding casualties were more likely to experience fatal injury severity. This could be attributed to the fact that speeding was the identified cause in most motorcycle crashes in this study, which also saw a relatively high proportion of severe injuries. These findings stress the need to address speeding-related risks during specific periods to reduce severe and fatal motorcycle crash injuries. Further research should focus on the underlying factors causing these instabilities to inform targeted interventions aimed at mitigating the impact of speeding on motorcycle safety.

In 2015, 2017 rainy season models and the 2017 dry season model, improper overtaking was observed to significantly raise the risk of fatal injury (as shown in Table 8). This pattern may be explained by the fact that crashes involving motorcyclists who engage in improper or illegal overtaking often led to severe accidents, such as head-on or high-speed collisions. This aligns with findings from previous research, which identified improper overtaking as a significant factor in motorcycle crash fatalities (Kashani et al., 2012). The observed temporal instability and the elevated risk during holiday periods underscore the need for targeted interventions and public awareness campaigns to promote safe overtaking practices. Future research could delve further into the specific contributing factors to improper overtaking incidents and explore the effectiveness of potential countermeasures to improve motorcycle safety.

Lastly, the alcohol usage indicator was found to significantly raise the probability of fatal injuries only in the 2015 rainy and dry seasons, as well as the 2017 rainy and dry seasons. This finding aligns with previous studies, which have indicated that alcohol consumption generally increases the injury severity in motorcycle crashes [19,34,109]. Targeted interventions should be implemented to reduce alcohol-related incidents both in rainy and dry seasons. A comprehensive approach involving public awareness campaigns, law enforcement, and community-based initiatives could prove effective in mitigating the adverse effects of alcohol consumption on motorcycle safety.

### 6.3 Roadway-related characteristics

The national road variable consistently increased the probability of fatal injuries in all three-year models, with the dry season models showing the largest increase in injury severity (as indicated in Table 8). Despite offering superior riding conditions and safety features compared to other road types, national roads continue to present a significant risk of fatal injuries for motorcyclists, largely due to their high speed limits [97,102,110]. Therefore, it is crucial to consider road types and their associated speed limits in assessing motorcycle crash injury outcomes. While national roads may offer better overall conditions, their higher speed limits can significantly contribute to more severe injuries in the event of a crash. Road safety interventions should consider the unique risks posed by different road types and aim to improve safety measures on all road categories to ensure better protection for motorcyclists and other road users. Further research could explore additional factors that might contribute to the observed temporal instability and differences in injury severity across different road types to develop more targeted safety strategies.

The major road variable stably and significantly increased the likelihood of fatal injuriy in 2015 rainy season and 2016 dry season models (as depicted in Table 8). In Cambodia, major roads refer to roads located within the city with double central lines, distinguishing them from national or provincial roads. One possible explanation for this finding may be the increased challenges in controlling motorcycle speed during this time. This result aligns with intuition and is consistent with previous studies [81,92]. Given the significance of this indicator in these two models, greater efforts should be directed towards improving motorcycle safety on major roads. These results highlight the importance of considering road types and their specific characteristics when assessing motorcycle crash injury outcomes. The significance of crashes on major roads suggests the need for targeted interventions and safety measures on these road segments to reduce the risk of fatal injuries for motorcyclists. Further research could delve deeper into the contributing factors to these crashes on major roads and evaluate the effectiveness of potential countermeasures to enhance motorcycle safety.

In all seasonal and yearly models, urban areas (as opposed to rural areas) significantly decreased the probability of fatal injury (as shown in Table 8). Generally, motorcycle crashes in urban environments are less severe than those in rural areas, and several factors contribute to this difference. These include lower speed limits in urban settings, dense traffic volumes that discourage high-speed motorcycle operation, better surrounding built environment in urban areas, and a higher tendency among urban riders to use safety helmets [111,112]. This finding provides valuable insights into the safety dynamics of motorcycle crashes in different environments. Urban areas appear to offer certain advantages in terms of reducing the severity of injuries sustained in motorcycle crashes. Such insights could inform targeted road safety interventions and policies to further improve motorcycle safety, especially in rural regions where crash severity tends to be higher. By addressing the specific challenges faced in rural areas and promoting safe riding practices, we can work towards reducing the impact of motorcycle crashes on casualties and fostering safer road environments for all road users.

### 6.4 Temporal characteristics

The variable related to motorcycle crashes occurring during weekends showed statistical significance in only some of the rainy season and dry season models. As presented in Table 8, the average marginal effect of Weekend indicated an increase in the likelihood of fatal injury in the 2015 rainy season, 2016 rainy season, and 2017 rainy season. There are several possible explanations for this observation. One possible explanation could be that weekends act as an incentive for risk-seeking behaviors among motorcyclists, which could subsequently increase the injury severity [24,55]. Additionally, riders might increase their speed to compensate for time lost due to riding at lower speeds during times of high traffic volume, which also potentially increases the injury severity. The variability in the effect of the weekend indicator may be influenced by factors such as increased traffic volumes during weekends due to local people engaging in travel activities. These factors could contribute to different outcomes in crash severity levels, depending on specific circumstances and road conditions during weekends. Overall, the findings suggest that weekends may represent a critical period for motorcycle safety, given the increased likelihood of fatal injuries during these times. To improve motorcycle safety during weekends, road safety measures could focus on addressing risk-taking behaviors, promoting responsible riding practices, and increasing awareness of potential hazards during high traffic periods. Further research could explore the underlying factors contributing to the weekend effect on motorcycle crashes to develop targeted interventions for enhancing motorcycle safety during these specific time periods.

### 6.5 Random parameters and heterogeneity in means and variances

**Fig. 2:**
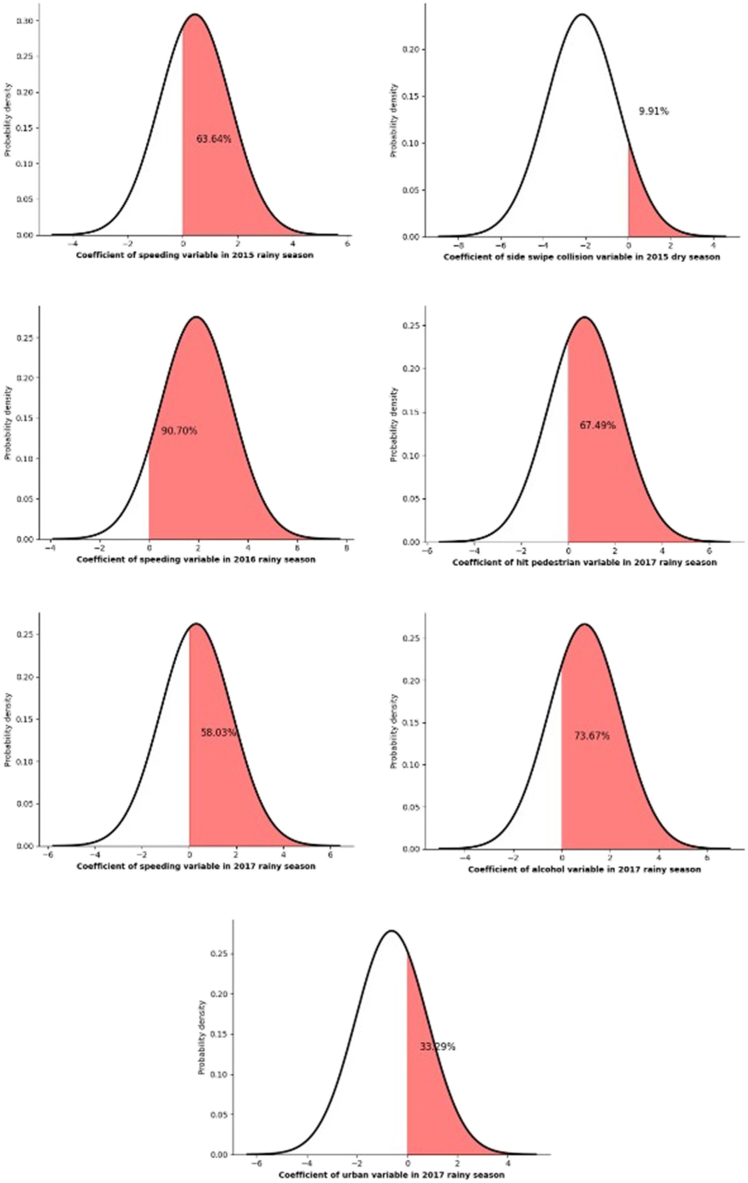
Random parameters related to fatal injury severity of the RPLMV models.

Beginning with random parameters estimation results, as shown in Table 7 and Fig. 2, in the 2015 rainy season model, statistically significant random parameters were observed for casualties due to speeding, with μ = 0.451 and σ = 1.672. According to the normal distribution used in our model for the random parameters, this result indicates that 63.64 % of the casualties due to speeding in the 2015 rainy season sample data were more likely to experience fatal injury severity. While 90.70 % and 58.03 % of the casualties who were speeding related to fatal injury severity in 2016, 2017 rainy season models. In the 2015 dry season model, 9.91 % of the casualties involved in the side swipe collisions were more likely to sustain fatal injury severity. Results showed that the hit pedestrian, speeding, alcohol, and urban indicators produced significant random parameters for the 2017 rainy season model. Specifically, this means that 67.49 % of the casualties involved in hit pedestrian crashes in the 2017 rainy season dataset were more prone to sustain fatal injury severity. Additionally, 73.67 % of the casualties involved in drinking alcohol behavior, and 33.29 % of the casualties involved in urban crashes had a higher likelihood of experiencing fatal injury severity. Overall, these statistical findings provide valuable insights into the random factors influencing injury severity in motorcycle crashes across different indicators and time periods, enhancing our understanding of motorcycle safety and the potential for targeted interventions to reduce injury severity in specific scenarios.

Regarding heterogeneity in means, the influence of side swipe crashes was decreased by the hit pedestrian indicator in 2015 rainy season model. And in the same model, the hit pedestrian indicator notably reduced the mean values of the alcohol indicators. In terms of heterogeneity in means in 2017 rainy season model, the presence of a male indicator was found to increase the mean of both the speeding and alcohol indicators. This increase suggests a higher likelihood of fatal injuries occurring in scenarios involving these factors. Regarding heterogeneity in variance, the urban indicator demonstrated significant variability in its variance, especially noticeable in cases of side swipe collisions, where it resulted in an increased variance of random. In terms of heterogeneity in means, the impact of wearing a helmet during crashes showed variation depending on the type of collision. Specifically, in right-angle collisions, wearing a helmet was associated with a decrease in the mean of random parameters, suggesting a reduced likelihood of fatal injuries. Conversely, in collisions involving hitting a pedestrian, helmet use was linked to an increase in the mean of random parameters, indicating a higher likelihood of sustaining fatal injuries. It may be because of the risk compensation behavior when wearing a helmet. The rider may feel safer when wearing a helmet, resulting in a more severe injury severity. Additionally, the effect of national road crashes was found to vary by head-on collision and fall alone collision (decreasing the mean of random parameters, rendering fatal injury less likely). Regarding heterogeneity in variance, for the 2017 dry season model, the hit passenger vehicle indicator and urban indicator produced significant random parameters, indicating that 70.78 % of the casualties involved in hit passenger vehicle crashes were more likely to sustain fatal injury severity, while 44.79 % of the casualties involved in urban crashes were more likely to sustain fatal injury severity. In terms of mean heterogeneity, the hit pedestrian and hit motorcycle indicators increased the likelihood of fatal injuries in weekend and speeding collisions, respectively. These findings highlight the variability and complexities in the relationship between different indicators and injury severity in motorcycle crashes, emphasizing the importance of considering various factors when analyzing crash data and designing safety interventions for motorcyclists.

## 7 Policy recommendations

This section aims to offer immediate recommendations for improving motorcyclist safety. Policymakers should focus on factors that notably increase the risk of severe injuries to motorcyclists, especially those showing worsening trends over time. Addressing these issues is key to reducing motorcycle-related injuries and fatalities.

In all rainy season and dry season models, head on collisions increase the fatal injury severity with the largest marginal effects. Therefore, riders should pay more attention to avoid head on collision. Additionally, elderly casualties suffer a higher likelihood of sustaining fatal injuries, so specific education and campaigns should be deployed to target elder riders during these periods.

In rainy season crashes, the indicator of hit motorcycle has the largest marginal effects on severe injury severity in the rainy season than that of the dry season. Therefore, more attention should be paid to hit motorcycle crashes during rainy seasons in order to reduce severe injury severity. Motorcycle riders should exercise greater caution when riding around other motorcycles in rainy seasons. Furthermore, the weekend factor consistently increases the risk of fatal injuries to motorcyclists in all three-year models for rainy seasons, showing temporal stability. Hence, it is crucial to focus on enhancing motorcycle safety during weekends in rainy seasons, as this period poses a heightened risk for motorcyclists.

Similarly, in dry season crashes, the indicators of being the rider and national road have the largest marginal increase in the probability of fatal injury severity compared to the other variables in the dry season. Hence, targeted safety awareness campaigns for motorcycle riders are essential, especially on national roads during rainy seasons, to prevent fatal injuries.

Among the significant crash characteristic factors, many increase the risk of more severe injuries to motorcyclists, though some exhibit declining trends over time, suggesting improvements in motorcyclist safety due to related policies. This indicates the effectiveness of current policies in either exacerbating or ameliorating motorcyclist safety within the transportation system, particularly in developing Southeast Asian countries like Cambodia. The mitigatory effect of wearing a helmet should also be noted as it significantly reduces the probability of severe and fatal injury severity. Therefore, helmet regulations should be maintained. Motorcyclist casualties involved in urban area crashes have a lower likelihood of suffering from fatal injury severity, indicating that developing local roadway infrastructures could significantly help in reducing fatal injuries.

It should be emphasized that factors with significant temporal variability may not offer clear guidance for long-term planning, necessitating more comprehensive investigations. For stable long-term policymaking, it's crucial to regularly update plans based on factors showing distinctive temporal effects. Further in-depth studies are needed to unravel their complexities and understand their implications for motorcyclist safety.

## 8 Summary and conclusions

Motorcycle crashes continue to pose a major threat for road safety, given their high fatality rates compared to non-motorcycle crashes, especially in developing countries like Cambodia. This study adopts a novel approach by analyzing how rainy and dry season crashes differently impact motorcyclist injury severity, based on motorcycle crash data from Cambodia between 2015 and 2017, while also considering transferability and temporal shifts. The analysis employs advanced methodological techniques, specifically a random parameters logit model with heterogeneity in means and variances, to address unobserved heterogeneity and varying levels of injury severity (minor, severe, and fatal). Compared with the other three models, the incorporation of random parameters with heterogeneity in means and variance led to a marked improvement in statistical performance. The Likelihood ratio tests revealed that models for each season were not transferable across years, indicating considerably no transferability and temporal instability across all time-of-year models. Moreover, numerous statistically significant factors were identified as influencing the probabilities of motorcyclist injury severity across different seasons and in various yearly models, highlighting the complexity and variability of factors affecting motorcyclist safety.

A number of variables generate significant effects on motorcyclist severe and fatal injury probabilities both in rainy season and dry season, like hit motorcycle, hit pedestrian, elder riders, wearing helmet, urban, national road factors. While the weekend and overtaking variable only significantly increase the fatal injury severity in rainy season. These findings highlight the importance of implementing stricter penalties and educational programs to curb risky behaviors, such as riding without a helmet or by elderly riders, during both rainy and dry seasons. It is crucial to design season-specific strategies to target these behaviors effectively.

Regarding the assessment of temporal stability, only three variables (hit motorcycle, hit pedestrian, helmet, and national road) exhibited stability across all periods in the rainy season and dry season model, while other variables did not, indicating the effectiveness of current effort to reduce motorcycle crash and the need for more careful monitoring and understanding of these variations in the future. From a long-term perspective, considering this temporal stability, policy making recommendations like relevant educational initiatives, and effective enforcement countermeasures are suggested to decision makers.

Insights from random parameters with heterogeneity in means and variance indicates that there are several risk factors in interactions with other random variables. For instance, male riders tend to have high severe and fatal injury likelihood when they are speeding or drunk, etc. These findings shown in random parameters with heterogeneity in means and variances model reveal that potential interactions between risk factors exist, which would lead to over-estimated or under-estimated of potential risk factors if they are not considered properly.

However, limitations of this research such as self-selection issue [34,[113], [114], [115]], the potential misclassification or under-reported no injuries [70,116], and potential spatial correlations and instability issues etc. should be given better solutions in future research work.

Overall, this study underscores the critical need to account for seasonal temporal variations and transferability, along with unobserved effects, in assessing motorcyclist injury severity. The divergent outcomes observed between the rainy and dry seasons provide insights that can assist practitioners, researchers, institutions, and policymakers in improving road safety, especially for motorcyclists. These findings are particularly relevant for developing more effective safety policies and injury prevention strategies in regions like Southeast Asia, where motorcycle use is prevalent.

## CRediT authorship contribution statement

**Yaqiu Li:** Writing – review & editing, Writing – original draft, Software, Methodology, Formal analysis, Data curation, Conceptualization. **Junyi Zhang:** Writing – original draft, Investigation, Funding acquisition. **Haoran Li:** Software, Methodology. **Yunpeng Lu:** Writing – original draft, Data curation. **Lon Virakvichetra:** Data curation.

## Data availability statement

The data that has been used is confidential. The participants of this study did not give written consent for their data to be shared publicly, so due to the sensitive nature of the research, supporting data is not available.

## Declaration of competing interest

The authors declare that they have no known competing financial interests or personal relationships that could have appeared to influence the work reported in this paper.
